# Exploring the Potential of Lateral Wedge Insoles in Alleviating Bone Marrow Lesions in End-Stage Knee Osteoarthritis: A Preliminary Case Report

**DOI:** 10.7759/cureus.52473

**Published:** 2024-01-18

**Authors:** So Tanaka, Takanori Taniguchi, Masami Tokunaga, Takaaki Yoshimoto, Tomohiko Nishigami

**Affiliations:** 1 Department of Rehabilitation, Fukuoka Orthopedic Hospital, Fukuoka, JPN; 2 Faculty of Medical Science, Department of Physical Therapy, Fukuoka International University of Health and Welfare, Fukuoka, JPN; 3 Department of Orthopedics, Fukuoka Orthopedic Hospital, Fukuoka, JPN; 4 Faculty of Health and Welfare, Department of Physical Therapy, Prefectural University of Hiroshima, Hiroshima, JPN

**Keywords:** gait, co-contraction ratio, lateral wedge insole, varus thrust, bone marrow lesion, knee osteoarthritis/ koa

## Abstract

The efficacy of lateral wedge insoles (LWIs) in patients with end-stage knee osteoarthritis (OA) is unclear.

A 43-year-old male underwent two anterior cruciate ligament reconstructions in his right knee and was later diagnosed with end-stage knee OA. An LWI combining arch support with a lateral heel wedge was fabricated for this patient and used over 12 months. As a result, after 12 months, the bone marrow lesion (BML), as measured by the magnetic resonance imaging Osteoarthritis Knee Score (MOAKS), was downgraded from grade 2 to grade 1.

The use of LWI in a patient with end-stage knee OA showed lower co-contraction ratios in knee muscles even after 12 months. The results provide preliminary evidence suggesting the use of LWI in patients with end-stage knee OA has potential benefits for reducing BML.

## Introduction

Varus thrust is observed in 12% to 46% of patients with knee osteoarthritis (OA) [1], and its presence heightens the risk of OA progression [2]. Varus thrust is also associated with an increased risk of developing or exacerbating bone marrow lesions (BMLs) in the medial femorotibial joint over two years [3]. Factors affecting varus thrust during walking include age, body mass index, muscle strength, static knee alignment, and elevated levels of anterior and posterior knee joint muscle co-contraction [4-6].

The American Academy of Orthopedic Surgeons (AAOS) Clinical Practice Guideline strongly recommends that lateral wedge insoles should not be used for patients with knee OA [7]. However, some studies have concluded that plantar inserts such as lateral heel wedges (LHWs) and laterally wedged insoles (LWIs) effectively reduce varus thrust in the short and medium term [8]. Shimada et al. specifically noted the immediate effectiveness of LWI in alleviating varus thrust in patients with knee OA exhibiting mild deformity [9]. However, the impact of insoles on reducing BMLs remains inconclusive.

We hypothesize that LWIs may offer long-term improvements in BMLs by mitigating varus thrust. In this proof-of-concept study, we discuss a case of post-traumatic end-stage knee OA, potentially demonstrating the benefits of LWI usage. This study serves as a foundation for future clinical trials aimed at establishing the efficacy of insole therapy for treating BMLs.

## Case presentation

Patient’s information

**Figure 1:**
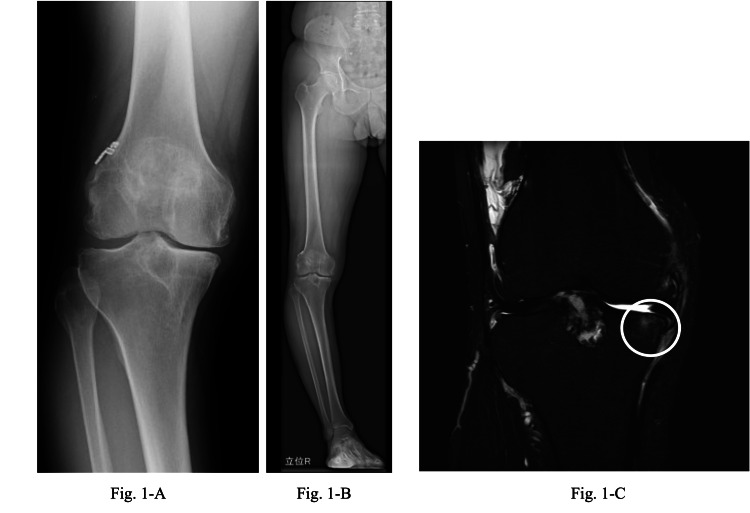
X-ray and MRI images of the patient before intervention. (A) X-ray: Medial knee OA (K-L grade 3; FTA: 187.5° in the right knee). (B) X-ray: %MA −13.1% in the right knee. (C) MRI: grade 2 BMLs were seen in the proximal medial part of the tibia. OA, osteoarthritis; MRI, magnetic resonance imaging; BML, bone marrow lesion; MA, mechanical axis; FTA, femorotibial angle; K-L, Kellgren-Lawrence

A 43-year-old Japanese male patient (height 173.2 cm; weight 72.0 kg) reported right knee pain persisting for over four years. The diagnosis indicated medial knee OA with a Kellgren and Lawrence grade of III. Further characterization revealed a femorotibial angle (FTA) of 187.5° and a mechanical axis deviation of -13.1% in the right knee (Figure 1). The patient's comfortable walking speed at 10 m was 8.4 seconds. The patient had no preexisting medical conditions other than knee OA.

The patient had undergone anterior cruciate ligament reconstructions in his right knee in 2001 and 2012 and was diagnosed with right knee OA in 2018. He was using 60 mg of nonsteroidal anti-inflammatory drugs (NSAIDs) intermittently for pain relief. In addition, he did not use any medications other than NSAIDs. In June 2021, following intense work, he experienced severe right knee pain. An orthopedic surgeon recommended a closed-wedge high tibial osteotomy for his severe knee deformity. Due to work commitments, he could not undergo surgery for another year and was thus prescribed an LWI for symptomatic relief and joint preservation. Before the intervention, the magnetic resonance imaging Osteoarthritis Knee Score (MOAKS) indicated a grade 2 BML in the medial femorotibial joint (Figure 1C). The pain and functional outcomes are outlined in Table 1. A varus thrust in the right knee was visibly evident during gait. The patient was informed about the study’s objectives and provided informed consent for the intervention. In addition, approval for this study was obtained from the institutional review board (IRB) of the hospital (approval number 2023-18).

**Table 1:** Outcome variables. Comparison of preintervention and 12 months postintervention. FTA, femorotibial angle; BML, bone marrow lesion; VAS, visual analog scale; KOOS, Knee Injury and Osteoarthritis Outcome Score

Factor	Preintervention	12 months postintervention
FTA (°)	187.5	187.6
BML (grade)	2	1
Pain at rest: VAS (mm)	0	0
Pain at motion: VAS (mm)	19	8
KOOS: Symptom	42.9	46.4
KOOS: Pain	78.1	82.4
KOOS: ADL	88.2	85.3
KOOS: Sports	35.0	35.0
KOOS: QOL	43.8	43.8
10 m gait speed (seconds)	8.4	7.9

Proof-of-concept trial

**Figure 2:**
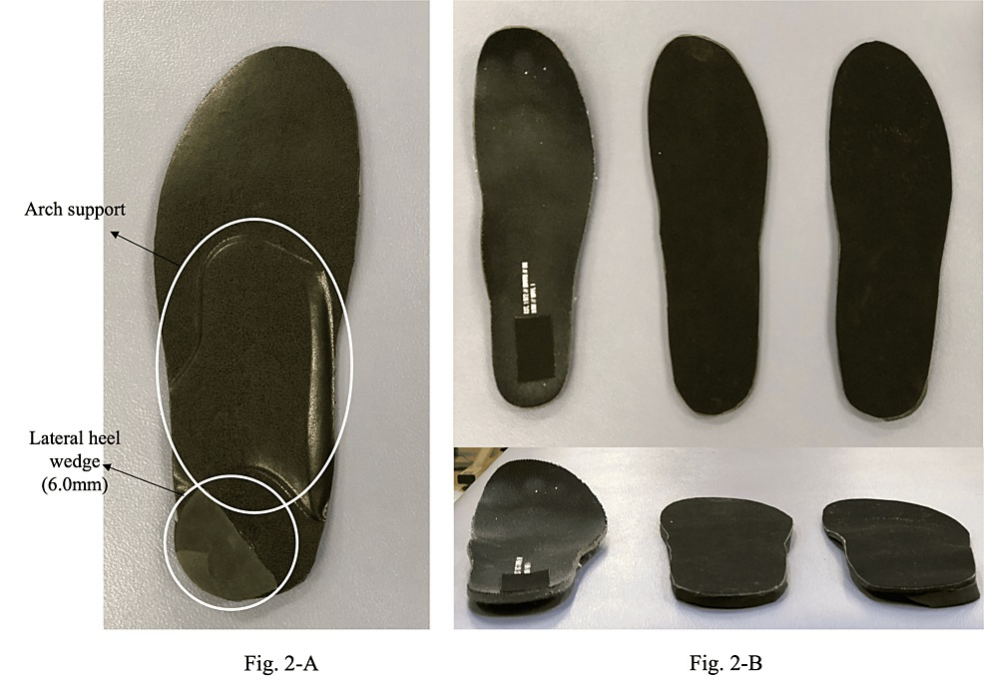
Each type of insole was used in this study. (A) LWI combining arch support and LHW; (B) from left to right: control, placebo, and LWI. Image credit: So Tanaka. LWI, lateral wedge insole; LHW, lateral heel wedge

We designed an insole that combines an LHW with additional arch support, as studies have reported its greater effectiveness compared to using an LHW alone in reducing varus thrust (Figure 2A) [10]. The arch support was customized in height and shape to accommodate free gait. In addition, the LHW had a minimum height of 6.0 mm at which varus thrust was most effectively attenuated, as two physiotherapists independently assessed the patient’s gait [11].

Measurements were conducted under three conditions for a single case: (1) a control insole (insole that came with the shoe), (2) a flat insole of identical thickness to the LWI (placebo), and (3) the LWI itself (Figure 2B). All conditions were evaluated on a treadmill at a comfortable walking speed (2.5 km/hour) and rhythm (84 beats per minute [BPM]). In addition, the participants were asked to walk on a treadmill to replicate their usual gait without specific instruction on posture during walking. Each condition was tested three times, and patients were blinded to the type of insole used.

A three-dimensional motion analyzer, Myomotion by Noraxon, Scottsdale, AZ, was employed to measure varus thrust. Accelerometers were attached to the bilateral dorsal foot and tibial tuberosities, and the peak varus thrust immediately after initial contact (IC) was quantified as thrust acceleration (TA) [12]. Myomuscle (Noraxon) measured muscle activity during walking, and the co-contraction ratio (CCR) affecting varus thrust was calculated. The muscles examined included the vastus medialis, vastus lateralis, semitendinosus, and biceps femoris on the affected side. CCRs for both medial and lateral muscles were calculated from IC to TA [6]. An accelerometer was also affixed to the sacral region to measure the acceleration of the sacrum in the anterior direction during walking.

**Figure 3:**
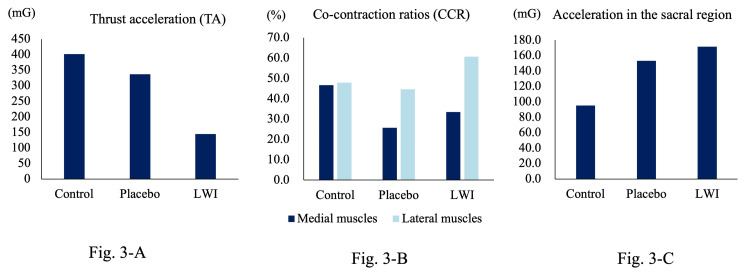
Comparison of anterior acceleration in the varus thrust, CCR, and sacral region under three conditions. (A) Comparison of TA under three conditions. (B) Comparison of CCR of medial and lateral muscles according to three conditions. (C) Comparison of acceleration in the anterior direction in the sacral region. LWI, lateral wedge insole

TA values were 402, 337, and 145 mg for the control, placebo, and LWI groups, respectively, with the lowest value observed in the LWI group (Figure 3A). Medial CCRs were 46.7%, 25.7%, and 33.5% in the control, placebo, and LWI groups, respectively (Figure 2D). The lateral CCRs were 48.0%, 44.6%, and 60.8% in the control, placebo, and LWI groups, respectively (Figure 3B). Furthermore, the acceleration in the anterior direction in the sacral region values were 95.2, 153.2, and 171.4 mG for the control, placebo, and LWI groups, respectively, with the highest value observed in the LWI group (Figure 3C).

Gait assessment using the three-dimensional motion analyzer confirmed a reduction in varus thrust. Accordingly, the LWI was worn for eight hours a day and 40 h a week for 12 months. The patient visited the orthopedic surgeon every three months, and the physiotherapist checked for LWI degradation and fitting compliance. No other treatments were administered during this 12-month period, and the patient’s activity level remained constant.

Patient follow-up

**Figure 4:**
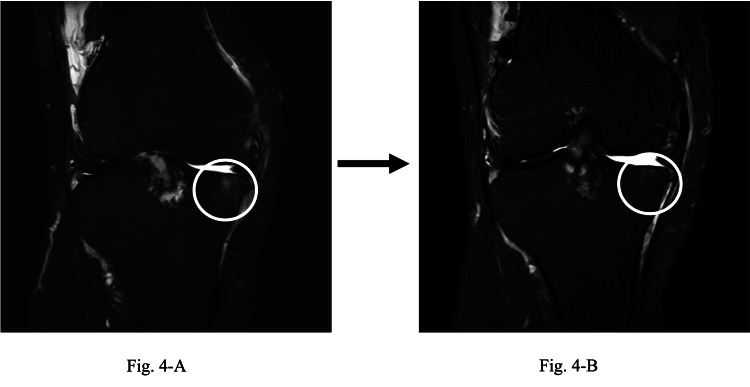
Comparison of BML size MRI before and after intervention. Change in BMLs. The BMLs, which were initially graded 2 (A), were reduced to grade 1 after 12 months (B). BML, bone marrow lesion; MRI, magnetic resonance imaging

A 12-month follow-up was conducted in coordination with an orthopedic surgeon’s visit. No significant changes in pain or function were observed compared to preintervention levels (Table 1). Lower extremity alignment measurements showed an FTA of 187.6°. Furthermore, BML in the medial femorotibial joint reduced from grade 2 at baseline to grade 1 at 12 months (Figure 4).

**Figure 5:**
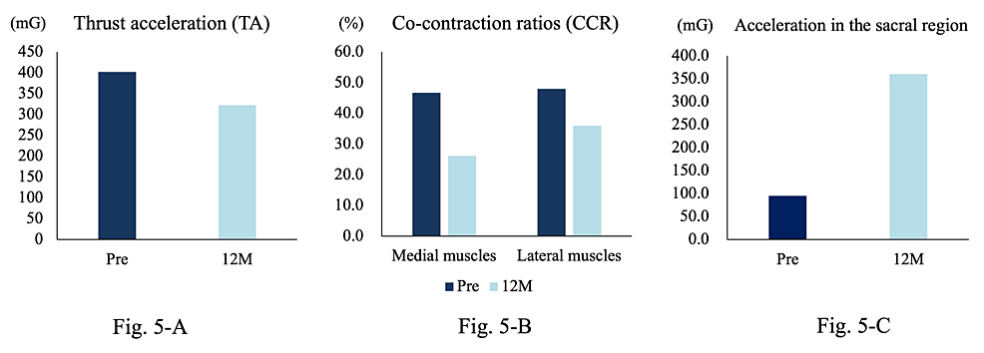
Results of varus thrust, CCR before and after intervention, and anterior acceleration of the sacral region. (A) TA was 332 mG at 12 months compared to 402 mG at baseline. (B) The initial medial CCR was 46.7%, decreasing to 26.2% at 12 months, while the initial lateral CCR was 48.0%, decreasing to 36.0% at 12 months. (C) The anterior acceleration of the sacral region increased from 95.2 mG at baseline to 360.2 mG at 12 months.

Motion analysis was conducted under identical conditions as at baseline, using the originally prescribed LWI inserts. The results revealed a reduction in TA from 402 mG at baseline to 332 mG at 12 months (Figure 5A). Initial medial CCRs decreased from 46.7% to 26.2%, and lateral CCRs decreased from 48% to 36% at 12 months (Figure 5B). In addition, the anterior acceleration of the sacral region increased from 95.2 mG at baseline to 360.2 mG at 12 months (Figure 5C).

## Discussion

This report documents a case in which the placement of an LWI resulted in a reduction of varus thrust and a downgrade of BML after 12 months. We initially hypothesized that LWI would reduce CCR, varus thrust, and BML more than placebo and control. Contrary to our hypothesis, both LWI and placebo attenuated CCR at baseline when compared to controls. This is likely attributed to the impact-reducing effect of insoles, achieved by increasing thickness [13]. Despite this, placebo insoles failed to mitigate varus thrust, prompting us to persist with LWI usage. Consequently, the indicator for varus thrust showed a reduction at 12 months, not at baseline. Additionally, CCR values at 12 months were lower than baseline values, both medially and laterally.

Concerning the variability of BMLs, the literature provides conflicting data. Roemer et al. reported that 50% of patients showed either regression or resolution at 30 months [14], while Hunter et al. found that 99% of patients exhibited the same or increased BML size, with only 0.6% showing reduction [15]. Hunter et al. also reported that BML scores are likely to increase in the presence of marked internal alignment, especially in patients with 7° to 23° varus alignment [15]. Herein, due to the 7.5° varus alignment of the case, the BMLs were more likely to remain the same or increase. Weight, often a variable that could influence BMLs, remained constant from baseline to follow-up. Thus, we speculate that LWI contributed to the BML reduction. While 60% to 80% of knee joint forces are primarily generated by muscle forces [16], increased CCR increases joint compression, which, in turn, affects OA progression [17]. Specifically, elevated medial muscle CCR has been reported in patients with end-stage knee OA [18], and higher medial-to-lateral CCR ratios contribute to cartilage loss. Therefore, our patient’s medial CCR reduction may indicate a potential mechanism for inhibiting OA progression [18].

In addition, an increase in forward acceleration of the sacral region during walking was observed after the intervention compared to before the intervention. It has been reported that sacral forward acceleration is lower in patients with knee OA compared to healthy subjects [19]. However, in this study, sacral anterior acceleration showed an increase both immediately and after 12 months. This was thought to result from a smoother forward movement of the pelvis in the early stance phase due to the weakening of the varus thrust.

This study is limited by its single-case nature, and it is unclear if there is a causal relationship between varus thrust, CCR, and BMLs. While BMLs naturally exhibit variability [14,15], orthotic interventions have been reported to reduce BMLs in the patellofemoral joint [20]. These preliminary findings suggest that mechanical load reduction may contribute to BML reduction and warrant further investigation to validate the efficacy of LWI in patients with post-traumatic knee OA.

## Conclusions

We discuss the efficacy of an LWI in mitigating varus thrust in patients with post-traumatic end-stage knee OA. We found that LWI led to prompt attenuation of varus thrust and reduced CCR in both anterior and posterior knee muscles. This attenuation effect persisted for 12 months, suggesting it might play a role in preventing BML reduction in the medial femorotibial joint. These initial observations require validation in subsequent clinical trials to confirm the effectiveness of LWI in patients with post-traumatic end-stage knee OA.
